# Predictive Features and Clinical Presentation of Interstitial Lung Disease in Inflammatory Myositis

**DOI:** 10.1007/s12016-020-08814-5

**Published:** 2020-11-03

**Authors:** Tamara Vojinovic, Ilaria Cavazzana, Paolo Ceruti, Micaela Fredi, Denise Modina, Marialma Berlendis, Franco Franceschini

**Affiliations:** 1 Rheumatology and Clinical Immunology Unit, ASST Spedali Civili, Piazzale Spedali Civili 1, 25123 Brescia, Italy; 2 Pulmonology Unit, ASST Spedali Civili, Piazzale Spedali Civili 1, 25123 Brescia, Italy; 3 Clinical and Experimental Science Department, University of Brescia, Piazza del Mercato 15, 25121 Brescia, Italy

**Keywords:** Idiopathic inflammatory myositis (IIM), Interstitial lung disease (ILD), Anti-Ro52 antibodies, Anti-ku antibodies, Anti-synthetase syndrome

## Abstract

Interstitial lung disease (ILD) represents one of the most severe extra-muscular features of idiopathic inflammatory myositis (IIM). We aimed to identify any clinical and serological predictors of ILD in a monocentric cohort of 165 IIM patients.

ILD+ patients were defined as having restrictive impairment in lung function tests and signs of ILD at chest high-resolution computed tomography (HRCT). Available HRCT images were centralized and classified in different ILD patterns: non-specific interstitial pneumonia (NSIP), organizing pneumonia (OP), usual interstitial pneumonia-like (UIP), indeterminate for UIP, and interstitial lung abnormalities (ILA). Lung function test data were recorded at onset, at 1 and 5 years after ILD diagnosis.

ILD was found in 52 IIM patients (31.5%): 46.2% was affected by anti-synthetase syndrome (ARS), 21% by polymyositis (PM), 19% by dermatomyositis (DM), and 13.5% by overlap myositis. Most of ILD+ showed NSIP (31.9%), OP (19%), indeterminate for UIP (19%), and UIP (12.8%) patterns. At multivariate analysis, ILD was predicted by anti-Ro52 (*p*: 0.0026) and dyspnea (*p*: 0.015) at IIM onset. Most of ILD onset within is 12 months after IIM. In five cases, ILD occurs after 12 months since IIM diagnosis: these patients more frequently show dry cough and anti-Ku antibodies. Anti-Ro52 + ILD patients showed a significant increase of DLCO at 1 and 5 years of follow-up, compared with anti-Ro52 negative cases.

ILD occurs in about one third of IIM and was predicted by dyspnea at onset and anti-Ro52 antibodies. Anti-Ro52 defines a subgroup of ILD showing a significant improvement of DLCO during follow-up.

This retrospective study has been approved by local ethic committee (ASST-Spedali Civili of Brescia, Italy); protocol number: NP3511

**Electronic supplementary material:**

The online version of this article (10.1007/s12016-020-08814-5) contains supplementary material, which is available to authorized users.

## Introduction

Interstitial lung disease (ILD) represents one of the most frequent complaints in idiopathic inflammatory myositis (IIM) and could deeply mark the disease course, in terms of functional limitation, treatment strategy, and mortality [1]. The rate of ILD widely ranges from 20 to 86%, depending on the screening technique used and the composition of different types of IIM considered [2]. ILD is one of the clinical hallmarks of anti-synthetase syndrome (ARS) [3], characterized by the triad of myositis, arthritis, and ILD, in which ILD occurs in about 60–80% of cases [4]. In non-Jo-1+ ARS, ILD is diagnosed in up to 80% of cases and could even represent the first symptom, at disease onset, defining a group of patients with a worse prognosis compared with anti-Jo-1+ cases [4]. Nevertheless, the prognosis of ILD-associated ARS is usually favorable [5], with improvement of lung function tests and a good response to treatment [6]. By contrast, a severe ILD, named rapidly progressive ILD (RPILD), was described, especially in Asian cohorts, in hypo- or amyopathic dermatomyositis (DM), associated with anti-MDA-5 antibodies, characterized by a rapid decline of functional status and a high mortality rate [6–11].

Although many papers define the rate and the radiological patterns of ILD associated with IIM [1, 4], few data are published regarding predictive factors of ILD development and its prognosis [1, 6, 7, 12, 13], especially in Caucasian cohorts.

The aim of this retrospective study was to identify clinical and serological features of IIM patients occurring at onset, able to predict the early or late development of ILD during follow-up.

## Patients

We retrospectively analyzed 165 consecutive adult patients affected by IIM, followed up in Rheumatology and Clinical Immunology Unit of Brescia, Italy, in order to identify patients with ILD. Most of IIM were represented by polymyositis (PM) (52 cases), DM (55 cases), or overlap IIM (24 cases), according to the Bohan and Peter criteria [14]; ARS [15] were found in 31 patients, while inclusion body myositis (IBM) [16] and necrotizing autoimmune myositis (NAM) [17] were diagnosed in 2 and 1 patients, respectively. A diagnosis of IIM in overlap with other systemic autoimmune diseases was made when recent criteria for systemic sclerosis (SSc) [18], systemic lupus erythematosus (SLE) [19], Sjogren’s syndrome (SS) [20], and rheumatoid arthritis (RA) [21] were achieved.

Clinical data were obtained from clinical charts. For all the patients, we collected demographic and epidemiological data; extramuscular findings, including skin manifestations (DM rash, mechanic’s hands, sclerodactyly, ischemic ulcers), calcinosis, arthritis, Raynaud’s phenomenon, dysphagia, myocarditis; myositis, defined as loss of muscle strength, myopathic electromyographic alterations, elevations of CK, transaminases and LDH values at onset, and histological findings in muscle biopsy, when performed.

Lung involvement was clinically defined as dyspnea and/or dry cough or occasional finding of ILD at thoracic imaging. According to clinical practice, at IIM onset, every patient performed chest X-ray and lung function test (LFT): if ILD was suspected, basing on symptoms, X-ray or LFT, a chest high-resolution computed tomography (HRCT) was performed to identify patients with ILD (i.e., ILD+). ILD+ patients were defined as having restrictive impairment in lung function tests (total lung capacity (TLC), forced vital capacity (FVC), and diffusing capacity of the lung for carbon monoxide (DLCO) < 80% of predicted) and signs of ILD at HRCT [22]. Isolated reduction of respiratory muscle strength, without any radiological finding of ILD, was excluded from ILD+ group. Basing on these data, ILD was diagnosed in 52 patients.

According to our clinical practice, ILD+ patients performed LFTs every year. In ILD+ group, we collected lung function test data at onset, at 1 year, and 5 years after ILD diagnosis. Clinical ILD onset was classified according to other authors [2, 5] in subacute presentation, chronic presentation, or asymptomatic ILD. An acute presentation (or RPILD) was defined as a worsening of radiologic interstitial change with progressive dyspnea and hypoxemia within 1 month of the onset of respiratory symptoms [10, 11].

## Methods

At our first observation, every IIM patient underwent chest X-ray and lung function tests, including TLC, FVC, DLCO, maximal inspiratory, and expiratory pressure (MIP and MEP) evaluation, and HRCT was performed in cases suspected for ILD. Available HRCT images were centralized and evaluated by an expert pulmonologist (C.P.), in order to confirm and/or recognize different ILD patterns. The radiological findings were grouped into six main HRCT patterns: non-specific interstitial pneumonia pattern (NSIP), organizing pneumonia pattern (OP), NSIP/OP overlap pattern, usual interstitial pneumonia-like pattern (UIP), indeterminate for UIP pattern, and interstitial lung abnormalities pattern (ILA), according to the current definitions in the literature [23–25].

Autoantibodies’ positivity was retrieved from clinical charts. Briefly, anti-nuclear were analyzed in all sera by IIF on HEp-2 cells, while anti-extractable nuclear antigen (ENA) antibodies were searched by ELISA (ANA screen 9 EliSA, Euroimmun, Lubeck, Germany) and immunoblot (ANA-PROFILE3, Eurommun, Lubeck, Germany). Myositis-specific and myositis-associated autoantibodies (MSA and MAA) were detected, at onset, in every IIM patient, when anti-ENA resulted negative, using commercial Lineblot (Euroline Autoimmune Inflammatory Myopathies 15 Ag (IgG) Euroimmun, Lubeck, Germany) including recombinant human proteins for Mi-2 alpha, TIF1γ, MDA-5, NXP-2, SAE, Ku, PM-Scl75/100, SRP, Jo-1, PL-7, PL-12, EJ, and OJ. Anti-HMGCR antibodies were tested with QUANTA Lite HMGCR ELISA in 57 available samples of myositis patients [26, 27].

This retrospective study was performed according to the principles of the Declaration of Helsinki.

## Statistical Analysis

The variables were expressed as mean ± standard deviation (SD). The difference between groups was detected by Fisher exact test and Student’s *t* test or Mann-Whitney test, when appropriated, using Statview Software (5.0.1.0). Significant values were defined for *p* < 0.05. Survival rate was analyzed with Kaplan-Meier curve and Log-rank (Mantel Cox) test.

## Results

### Demographic, Clinical, and Laboratory Features

ILD was found in 52 patients among 165 IIM cases (31.5%), with a mean age at onset of 54.13 years (SD: 14.7) and a mean follow-up of 92.4 months (SD: 66.4). The prevalence of ILD in different IIMs is shown in Table 1: ILD was significantly more frequent in ARS (77.4%) compared with other IIMs (21.37%; *p* < 0.0001; OR: 12.6; 95CI: 4.9–32.28), while less frequently occurred in DM (*p*: 0.008) and in PM (*p*: 0.048).

Among 24 cases of overlap IIM (13 SSc, 6 SLE, 3 Sjogren’s syndrome and 2 RA), 7 patients showed ILD: 4 IIM/SSc, 2 IIM/SLE, and 1 IIM/RA.

**Table 1:** ILD prevalence in different IIMs

	IIM-ILD (%)	*p*	OR
ARS	24/31 (77.4)^a^	< 0.0001	12.6 (4.9–32.28
DM	10/55 (18.2)^b^	0.008	0.34 (0.15–0.65)
PM	11/52 (21.1)^c^	0.048	0.45 (0.2–0.97)
Overlap IIM	7/24 (29.2)	0.8	

Not included 2 patients with inclusion body myositis (IBM) and 1 patient with necrotizing autoimmune myositis (NAM)

^a^ARS vs all

^b^DM vs all

^c^PM vs all

Demographic data of 52 ILD+ and 113 ILD− patients are summarized in Table 2. We recorded five deaths in ILD cases (9.6%), due to respiratory failure only in two: one case affected by PM, deceased after 13 years from onset, one case affected by overlap PM/SSc, deceased after 4 years from onset. No difference in survival rate was recorded between ILD+ and ILD− groups (*p*: 0.76; 100% at 1 year and 96% at 5 years in ILD+; 98% at 1 year and 95.5% at 5 years in ILD−).

**Table 2:** Demographic data of 52 IIM patients with ILD (ILD+) and 113 IIM patients without ILD (ILD−)

	ILD+ *n* 52 (%)	ILD− *n* 113 (%)	*p*
Age at onset (mean, SD)	54.13 (14.7)	50.45 (17)	0.18
Age (mean, SD)	61.89 (14.2)	60.8 (13.6)	0.63
Follow-up, months (mean, SD)	92.44 (66.4)	110.6 (96.7)	0.22
F/M *n* (ratio)	38/14 (2.7/1)	85/28 (3/1)	0.8
Caucasian	47 (90.4)	108 (95.6)	0.29
Deaths	5 (9.6)	15 (13.3)	0.61

ILD+ patients were more frequently affected by ARS (*p* < 0.0001; OR: 12.9) and less frequently by DM (*p*: 0.012) (Table 3). According to these data, ILD+ group showed more frequently anti-Jo-1 (*p* < 0.0001; OR: 5.6) and anti-MDA-5 antibodies (*p*: 0.033; OR: 7.05). On the contrary, anti-NXP2 are found only in ILD− cases (*p*: 0.033). Among MAA, anti-Ro52 antibodies were significantly associated with ILD (*p*: 0.0009; OR: 3.97) (Table 4). Among autoantibodies’ study, we registered 5 and 35 patients negative for MSA and/or MAA, respectively, in ILD+ and ILD− groups: these patients showed isolated ANA positivity without other autoantibodies’ specificity, including SSc-specific or SLE-specific serological markers.

**Table 3:** IIM distribution in 52 IIM patients with ILD (ILD+) and 113 IIM patients without ILD (ILD−)

	ILD pos *n* 52 (%)	ILD neg *n *113 (%)	*p*
ARS	24 (46.1)	7 (6.2)	< 0.0001^a^
DM	10 (19.2)	45 (39.8)	0.012^b^
PM	11 (21.1)	41 (36.3)	0.071
Overlap myositis	7 (13.5)	17 (15)	1
Necrotizing myositis	0	2 (1.8)	1
IBM	0	1 (0.9)	1

^a^OR: 12.98 (5.074–33.204)

^b^OR: 0.36 (0.164–0.789)

**Table 4:** Myositis-specific (MSA) and myositis-associated (MAA) autoantibodies in 52 IIM patients with ILD (ILD+) and 113 IIM patients without ILD (ILD−)

	ILD pos *n* 52 (%)	ILD neg *n* 113 (%)	*p*
Jo-1	23/52 (44.2)	14/113 (12.4)	< 0.0001^a^
PL7	3/23 (13)	3/69 (4.3)	0.163
PL12	3/23 (13)	2/69 (2.9)	0.098
OJ	2/23 (8.7)	0	0.06
MDA-5	4/23 (17.4)	2/69 (2.9)	0.033^b^
EJ	0/23	2/69 (2.9)	1
Mi2	2/23 (8.7)	13/69 (18.8)	0.341
SRP	2/23 (8.7)	4/69 (5.8)	0.63
SAE	1/23 (4.3)	0/23	0.25
HMGCoAR	0/16	1/41 (2.4)	1
NXP-2	0/23	12/69 (17.4)	0.033^c^
SMN	0/23	1/69 (1.4)	1
TIF1γ	0/23	7/69 (10.1)	0.186
Negative	5/52 (9.6)	35/113 (31)	0.003
Ro	5/52 (9.6)	9/113 (8)	0.76
Ro-52	13/23 (56.5)	17/69	0.0009^d^
Ku	4/52 (7.7)	9/113 (8)	1
U1RNP	5/52 (9.6)	4/113 (3.5)	0.142
PM/Scl	7/52 (13.5)	12/113 (10.6)	0.6

^a^OR: 5.6 (2.56–12.26)

^b^OR: 7.05 (1.19–41.49)

^c^OR: 0.000 (inf)

^d^OR: 3.97 (1.48–10.7)

No differences were detected in clinical features at IIM onset between groups, except for mechanic’s hands (*p* < 0.0001), arthritis (*p*: 0.01), polyarthritis (*p*: 0.001), and dyspnea (*p* < 0.0001) more frequently associated with ILD. By contrast, facial rash (*p*: 0.003), myositis (*p* < 0.0001), as well as higher levels of CK and AST, were more frequently found in ILD− IIM at onset, as shown in Table 5.

**Table 5:** Onset clinical features positively and negatively associated with ILD IIM at univariate analysis

	ILD pos *n* 52 (%)	ILD neg *n* 113 (%)	*p*	OR		
Mechanic’s hands	23 (44.2)	8 (7.1)	< 0.0001	10.4 (4.22–25.69)		
Arthritis	22 (42.3)	25 (22.1)	0.01	2.58 (1.27–5.235)		
Polyarthritis	16 (30.8)	10 (8.8)	0.001	4.58 (1.905–11)		
Myocarditis	4 (7.7)	2 (1.8)	0.079			
Dyspnea	46 (88.5)	50 (44.2)	< 0.0001	9.66 (3.8–24.4)		
Facial rash	10 (19.2)	49 (43.4)	0.003	0.311 (0.142–0.681)		
Myositis	32 (61.5)	102 (90.3)	< 0.0001	0.173 (0.075–0.398)		
CPK levels	1375.3 (1703.5)	4186 (6360.1)	0.0001			
AST levels	89.5 (85.7)	178.4 (250.1)	0.014			

Multivariate analysis confirmed that dyspnea at IIM onset as well as anti-52 kD Ro antibodies predict the development of ILD (*p*: 0.0155 and *p*: 0.0026, respectively), while facial rash and anti-NXP-2 antibodies were associated with no ILD development during follow-up (*p*: 0.055 and *p*: 0.0003, respectively).

Analyzing lung function test at baseline, at 1 and 5 years after ILD onset, we registered a significant improvement of FVC values between baseline and 1 year in a total of 52 ILD+ patients (*p*: 0.034) (Table 6). No difference was detected in DLCO, DLCO/VA, and FVC at baseline comparing different subgroups, namely, anti-Jo-1+, anti-Ro52+, anti-MDA5+, or anti-Ku+ patients. As shown in Table 6, 13 anti-Ro-52+ showed higher DLCO values at 1 and 5 years (*p*: 0.04 and 0.005, respectively) compared with 38 Ro52− patients. Anti-Jo-1 cases showed the same trend, with a significantly higher DLCO at 1 and 5 years (*p*: 0.049 and *p*: 0.021, respectively). When considering only anti-Jo-1 positive and negative cases without anti-Ro52 or Ro/SSA, this difference is not evident: isolated anti-Jo-1-positive and anti-Jo-1-negative groups showed the same values of FVC, DLCO, and DLCO/VA at baseline, at 1 and 5 years of follow-up.

**Table 6:** Variation of DLCO and FVC values during time in different autoantibodies’ groups in 52 ILD+

	DLCO baseline mean (SD)	DLCO 1 year mean (SD)	DLCO 5 years mean (SD)	FVC baseline mean (SD)	FVC 1 year mean (SD)	FVC 5 years mean (SD)
All ILD+ (*n* 52)	62.8 (22.9)	63 (18.4)	67 (17.5)	83.6 (21.4)	94.2 (23.8)^a^	92.7 (29)
all Jo-1+ (*n* 23)	58.3 (20.8)	56.8 (15.6)^b^	63.9 (20)	78.9 (20.9)	88.7 (26.8)	89.7 (31.9
Jo-1+ (Ro52−) (*n* 16)	55.1 (18.4)	55.58 (11.6)	57.5 (9.6)	75.9 (17)	90 (25.3)	89.55 (31.8)
MDA-5+	76.5 (16.38)	81.2 (22.4)	81.5 (27.5)	86.5 (10.5)	110 (23.6)	110 (12)
Ku+	78.7 (21.2)	78.5 (20.9)	61.3 (2.1)	93.7 (26)	105 (23.8)	83.3 (31.7)
Ro52+ (*n* 13)	70.7 (20.6)	73.5 (20.9)^c^	87 (19.6)^d^	89.8 (23.7)	80.4 (16.6)	92.6 (14.6)

^a^FVC baseline-1 year: *p* 0.034

^b^DLCO at 1 year: all Jo-1 vs other ILD: *p* 0.049

^c^DLCO 1 year: Ro52+ (73.5 ± 20.9) vs other ILD (59.5 ± 16.7): *p* 0.04

^d^DLCO 5 years: Ro52 (87 ± 19.6) vs other ILD (61.1 ± 11.4): *p* 0.0005

### ILD Features

Analyzing 52 patients with ILD, smoking habit was found in 8 patients (15.4%), while 44 patients (88.6%) were non-smokers. Clinical respiratory presentation was dyspnea (69.2%) and cough (21%), while no symptoms were reported in 21% of cases. A chronic ILD presentation was found in 30 patients (57.7%), subacute presentation in 5 (9.6%), RPILD in 10 (19.2%), and asymptomatic onset in 6 (11.5%). In one case, ILD onset was before the diagnosis of DM. We recorded five deaths among ILD+, in only two cases related to respiratory failure (PM and overlap PM/SSc): no difference between dead and alive patients was found, except for anti-OJ antibodies more frequently detected in dead (2/5:40%) than in alive cases (0%; *p*: 0.012).

We encountered ten cases of RPILD, four of which showed significant desaturation since the onset (affected by ARS in two, DM and PM in one case each). HRCT patterns were represented by BOOP (five cases), NSIP (two cases), and UIP-like (three cases). No differences in terms of other clinical features as well as autoantibody distribution, including anti-MDA-5 antibodies, were found between RPILD and non-RPILD.

Chest CT images were available in 47 out of 52 patients. The assessment of CT images led to the following pattern classification: NSIP pattern in 15 (31.9%), OP pattern in 9 (19.1%), NSIP/OP pattern in 4 (8.5%), UIP-like pattern in 6 (12.8%), indeterminate for UIP pattern in 9 (19.1%), and ILA pattern in 4 (8.5%).

No significant differences in demographic data, diagnoses’ distribution, clinical features at IIM onset, or MSA occurrence were found between the patterns. Significant features associated with CT patterns are summarized in Table 1 (Supplemental material): at onset, NSIP cases showed a lower FVC value (*p*: 0.02) and significantly lower DLCO value (*p*: 0.0004) than UIP-like cases. During the time, no significant variations of DLCO, FVC, or DLCO/VA values were recorded both in NSIP and UIP-like cases both at 1 and 5 years since onset.

Most of ILD onset occurred within 12 months from the IIM diagnosis (47/52 patients: 90.38%). No differences in terms of diagnoses, smoking habit (past or current), clinical features at onset, or clinical presentation of ILD were found between patients with ILD onset before or after 12 months since IIM. Cough was more frequently reported in patients with ILD onset after 12 months (*p*: 0.057). MSA were equally distributed in two groups, while anti-Ku antibodies were more frequently detected in patients with ILD onset later (40%) compared with patients with ILD onset earlier (4.2%) (*p*: 0.048; OR: 13; 95%CI: 1.3–130).

## Discussion

ILD is a frequent clinical feature of IIM [1] with a wide variation of clinical presentation and prognosis, especially for the rapid progressive ILD variant [3, 5–8]. The heterogeneity of ILD behavior during the time and the clinical heterogeneity of IIM itself make this feature difficult to manage. Moreover, the absence of ILD in IIM classification criteria [28] and the lack of clinical predictive factors of ILD development and progression make ILD a clinical challenge.

The pathogenesis of different IIMs is a complex picture, where different actors are involved, including genetic predisposition, environmental triggers, dysregulated innate and adaptive immune response, variable expression of alpha interferon inducible genes [2, 29], and inflammatory cytokines and chemokines [29]. It is widely known that PM and DM show different expression of lymphocytes, with a predominant CD8+ T cell-mediated myofiber infiltration in PM, while a B and CD4+ T cell infiltration in perivascular areas of muscle and skin is prevalent in DM [2].

T cell activation appears to play a key role also in initiating lung damage in both PM and DM-associated ILD, demonstrated by a predominance of T lymphocytes in bronchoalveolar lavage with a decreased CD4+/CD8+ ratio [30] and high inflammatory cytokine expression in ILD-IIM, in particular TNF alpha in ILD+ PM and DM. Different authors reported the high expression of IL-8 [31] and other inflammatory cytokines (namely, IL-15) in lung tissue of in MDA-5+ RPILD [32]. By contrast, although ARS represents the IIM most frequently associated with ILD, a specific link with a peculiar inflammatory chemokine expression remains speculative [2].

This study aimed to identify any clinical and immunological features predicting the development of ILD at IIM onset.

We recorded ILD in 31% out of 165 IIM, based on HRCT scans, reviewed by an expert pulmonologist, and lung function tests. ILD rate is higher in ARS, confirming previous data, reporting ILD in about 80% of ARS [1, 3, 5]. We found ILD in 19% of DM patients and this rate did not change when only CADM is considered: this data is partially in contrast with most of the published data reporting up to 80% of ILD in CADM in Asian cohorts [6].

Anti-MDA5 is usually associated with RPILD, also in some European and American cohorts [7, 8, 33]. In our case series, anti-MDA-5 antibodies were found in similar rate in both ILD+ and ILD− cases, as well as in acute and chronic presentation ILD+ cases. Anti-MDA-5 antibodies are considered the hallmark of RPILD with a poor outcome in Asian cohorts [6, 7]. In American cohorts, anti-MDA-5 antibodies were more rarely associated with ILD, and when occurred, ILD is usually responsive to immunosuppression [34], suggesting a peculiar behavior according to different ethic/genetic backgrounds. In our cohort, anti-MDA-5 did not represent a laboratory feature predicting the development of ILD: this discrepancy could be explained by different ethnic cohorts or by different laboratory methods used for detection, namely line blot in our case series, immunoprecipitation in Baltimora’s groups [34], and ELISA in other reports [6, 33]. Nevertheless, our data mirror those of a collaborative Italian study where ELISA, immunoprecipitation, and Western blot were used [35].

In our study, dyspnea at IIM onset and anti-Ro52 antibodies could predict the development of ILD (*p*: 0.0155 and *p*: 0.0026) by multivariate analysis. The finding that anti-Ro52 could be strictly associated to the development of ILD is well known. Anti-Ro52 antibodies are known to represent the myositis-associated antibody more frequently found in IIM, particularly in anti-Jo-1+ patients. Recently, different authors described that anti-Ro52 [36] or anti-Ro/SSA [37] represent a negative prognostic value due to their association with severe ILD picture, at onset. Nevertheless, both groups reported a progressive stabilization or improvement in ILD anti-Ro associated [36, 37], as reported in our cohort, where anti-Ro-52 is the only serological marker of functional tests’ improvement at 1 and 5 years after ILD onset. By contrast, other authors reported a higher rate of RPILD in anti-Ro52+ patients with ARS [38], while in juvenile DM, anti-Ro52 is a predictive factor of non-remission due to active associated-ILD [39].

The clinical presentation of ILD is variable in our cohort, spreading from asymptomatic to dyspnea and cough, as previously described [38]. Published data confirm that the different clinical presentations of IIM did not confer a predictive value of ILD severity [38]. Noteworthy, in our hands, the onset with severe myositis, characterized by higher CPK and AST levels, represented a protective clinical pattern from ILD, although by univariate analysis.

Most of ILD cases are onset early, namely, within 12 months from IIM: although anti-MDA-5 are found only in patients with early onset ILD, in our cohort, this autoantibody does not represent a marker of RPILD. In contrast, cough and anti-Ku antibodies were more frequently found in late onset ILD. Anti-Ku antibodies are a known marker of overlap disease, including IIM and systemic sclerosis (SSc), strictly associated with ILD [40]. Moreover, some authors defined a clinical picture characterized by anti-Ku positivity and elevated CPK, predictive of ILD development [41]. In our study, CK elevation and myositis are not associated with ILD; however, anti-Ku antibodies are associated with late onset ILD, namely after at least 12 months from IIM onset. This could suggest that a nonspecific symptom, such as cough, and a rare autoantibody, namely anti-Ku, are found in IIM patients with a late and slow onset of ILD. To date, it is unknown if anti-Ku could really mark ILD with a slow rate progression or if anti-Ku-positive patients could be misdiagnosed and achieve an ILD-IIM diagnosis definition later. Anyway, in our study, anti-Ku+ cases did not show difference in lung functional tests compared with anti-Ku-negative group, at baseline and at different timepoints during follow-up.

Different radiological HRCT patterns have been recorded in our IIM patients, according to other cohorts [1]: NSIP pattern represents the prevalent finding, while UIP-like and OP patterns were found in about 12% and 20% of cases. NSIP pattern is the prevalent CT pattern also in SSc, while UIP pattern covers about 10% of cases [42]. In SSc, UIP pattern defines patients with worse prognosis [43], as well as in ARS cases [44].

Patients with NSIP pattern onset with lower DLCO and FVC values compared with UIP-like cases. However, both groups remained stable during time, without significant reduction of functional parameters both at 1 and 5 years from onset.

Only four cases of ILA pattern were identified (8.5%). The exact meaning of this finding in autoimmune backgrounds is not yet known. However, recent data seem to suggest that these alterations share common genetically driven biologic pathways with idiopathic pulmonary fibrosis, which is a specific type of chronic, progressive, fibrosing interstitial pneumonia of unknown cause, with an unfavorable prognosis [45].

The present paper has some limitations, the most important of which is the retrospective design. Nevertheless, the long follow-up, the exhaustive search for autoantibodies, and the review of each HRCT available by an expert pulmonologist represent strength points of the paper.

## Conclusion

We report here a cohort of largely prevalent Caucasian patients affected from IIM from a single center. A third of patients developed ILD. ARS was significantly associated with ILD, while DM was negatively associated. Although dyspnea and anti-Ro52 antibodies are associated with ILD at multivariate analysis, anti-Ro52 antibodies define a subgroup of ILD-IIM with significant improvement during follow-up.

## Electronic supplementary material

Below is the link to the electronic supplementary material.

Supplementary file1 (DOCX 12.7 kb)

## References

[1] Harvier B, Uzunhan Y (2020). Inflammatory myopathy-related interstitial lung disease: From pathophysiology to treatment. Front Med.

[2] Saketkoo LA, Ascherman DP, Cottin V, Christopher-Stine L, Danoff SK, Oddis CV (2010). Interstitial lung disease in idiopathic inflammatory myopathy. Curr Rheumatol Rev.

[3] Cavagna L, Nuno L, Scire’ CA, Govoni M, Lopez-Longo FJ, Franceschini F (2015). Clinical spectrum time course in anti Jo-1 positive antisynthetase syndrome results from an international retrospective multicenter study. Medicine.

[4] Cavagna L, Trallero-Araguas E, Meloni F, Cavazzana I, Rojas-Serrano J, Feist E (2019). Influence of antisynthetase antibodies specificities on antisynthetase syndrome clinical spectrum time course. J Clin Med.

[5] Hu JW, Kim DS, Lee CK, Yoo B, Bum SJ, Kitaichi M, Colby TV (2007). Two distinct clinical types of interstitial lung disease associated with polymyositis-dermatomyositis. Respir Med.

[6] Yoshida N, Okamoto M, Kaieda S, Fujimoto K, Ebata T, Tajiri M (2017). Association of anti-aminoacyl-transfer RNA synthetase antibody and anti-melanoma differentiation-associated gene 5 antibody with the therapeutic response of polymyositis/dermatomyositis-associated interstitial lung disease. Respir Invest.

[7] Gan YZ, Zhang LH, Ma L, Sun F, Li YH, An Y, Li ZG, Yi H (2020). Risk factors of interstitial lung diseases in clinically amyopathic dermatomyositis. Chinese Med J.

[8] Labrador-Horrillo M, Martinez MA, Selva-O’Callaghan A, Trallero-Araguas E, Balada E, Vilardell-Tarres M, et al. (2014) Anti-MDA5 antibodies in a large Mediterranean population of adults with dermatomyositis. J Immunol Re 290797 10.1155/2014/290797 PMC3987881 24741583

[9] Moghadam-Kia S, Oddis CV, Sato S, Kuwana M, Aggarwal R (2017). Antimelanoma differentiation-associated Gene 5 antibody: Expanding the clinical spectrum in North American patients with Dermatomyositis. J Rheumatol.

[10] Chen Z, Cao M, PlanaZ, Liang J, Cai H, Kuwana M, Sun L. MN (2013). Utility of anti-melanoma differentiation-associated gene 5 antibody measurement in identifying patients with dermatomyositis and a high risk for developing rapidly progressive interstitial lung disease: a review of the literature and a meta-analysis. Arthr Care Res.

[11] Sato S, Hoshino K, Satoh T, Fujita T, Kawakami Y, Fujita T, Kuwana M (2009). RNA helicase encoded by melanoma differentiation-associated gene 5 is a major autoantigen in patients with clinically amyopathic dermatomyositis: Association with rapidly progressive interstitial lung disease. Arthritis Rheum.

[12] Zuo Y, Yie L, Liu M, Li S, Liu W, Chen F, et al (2020) Clinical significance of radiological patterns of HRCT and their association with macrophage activation in dermatomyositis. Rheumatology. 10.1093/rheumatology/keaa034 10.1093/rheumatology/keaa034 32065646

[13] Fathi M, Barbasso Helmers S, Lundberg IE (2012). KL-6: A serological biomarker for interstitial lung disease in patients with polymyositis and dermatomyositis. J Intern Med.

[14] Bohan A, Peter JB (1975). Polymyositis and dermatomyositis (first of two parts). N Engl J Med.

[15] Imbert-Masseau A, Hamidou M, Agard C, Groulleau JY, Cherin P (2003). Antisynthetase syndrome. Joint Bone Spine.

[16] Dimachkie MM, Barohn RJ (2012). Inclusion body myositis. Semin Neurol.

[17] Allenbach Y, Mammen AL, Benveniste O, Stenzel W (2018). Immune-Mediated Necrotizing Myopathies Working Group. 224th ENMC International Workshop: Clinico-sero-pathological classification of immune-mediated necrotizing myopathies Zandvoort, The Netherlands, 14–16 October 2016. Neuromuscul Disord.

[18] van den Hoogen F, Khanna D, Fransen J, Johnson SR, Baron M, Tyndall A (2013). Classification criteria for systemic sclerosis: An ACR-EULAR collaborative initiative. Arthr Rheum.

[19] Aringer M, Costenbaden K, Daikh D, Brinks R, Mosca M, Ramsey-Goldman R (2019). European League against rheumatism/American College of Rheumatology classification criteria for Systemic Lupus Erythematosus.

[20] Vitali C, Bombardieri S, Jonsson R, Moutsopoulos HM, Alexander EL, Carsons SE (2002). Classification criteria for Sjogren’s syndrome: A revised version of the European criteria proposed by the American-European Consensus Group. Ann Rheum Dis.

[21] Aletaha D, Teogi T, Silman AJ, Funovitis J, Felson DT, Bingham CO (2010). 2010 Rheumatoid arthritis classification criteria. Arhritis Rheum.

[22] Bradley B, Branley HM, Egan JJ, Greaves MS, Hansell DM, Harrison NK (2008). Interstitial Lung Disease Guideline. Interstitial lung disease guideline: The British Thoracic Society in collaboration with the Thoracic Society of Australia and New Zealand and the Irish Thoracic Society. Thorax.

[23] Travis WD, Costabel U, Hansell DM, King TE, Lynch DA, Nicholson AG (2013). An official American Thoracic Society/European Respiratory Society statement: Update of the international multidisciplinary classification of the idiopathic interstitial pneumonias. Am J Respir Crit Care Med.

[24] Sverzellati N, Lynch DA, Hansell DM, Johkoh T, King TE, Travis WD (2015). American Thoracic Society – European Respiratory Society Classification of the Idiopathic Interstitial Pneumonias: Advances in Knowledge since 2002. Radiographics.

[25] Raghu G, Remy-Jardin M, Myers JL, Richeldi L, Ryerson CJ, Lederer DJ (2018). Diagnosis of idiopathic pulmonary fibrosis. An Official ATS/ERS/JRS/ALAT Clinical Practice Guideline. Am J Respir Crit Care Med.

[26] Musset L, Miyara M, Benveniste O, Charuel JL, Shikhman A, Boyer O (2014). Analysis of autoantibodies to 3-hydroxy-3-methylglutaryl-coenzyme A reductase using different technologies. J Immunol Res.

[27] Musset L, Allenbach Y, Benveniste O, Boyer O, Bossuyt X, Bentow C (2016). Anti-HMGCR antibodies as a biomarker for immune-mediated necrotizing myopathies: A history of statins and experience from a large international multi-center study. Autoimmun Rev.

[28] Bottai M, Triamlund A, Santoni G, Werth VP, Pilkington C, de Visser M (2017). EULAR/ACR classification criteria for adult and juvenile idiopathic inflammatory myopathies and their major subgroups: A methodology report. RMD Open.

[29] Ceribelli A, De Santis M, Isailovic N, Gershwin NE, Selmi C (2017). The immune response and the pathogenesis of idiopathic inflammatory myositis: A critical review. Clin Rev Allergy Immunol.

[30] Englund P, Wahlström J, Fathi M, Rasmussen E, Grunewald J, Tornling G (2007). Restricted T cell receptor BV gene usage in the lungs and muscles of patients with idiopathic inflammatory myopathies. Arthritis Rheum.

[31] Gono T, Kaneko H, Kawaguchi Y, Hanaoka M, Kataoka S, Kuwana M (2014). Cytokine profiles in polymyositis and dermatomyositis complicated by rapidly progressive or chronic interstitial lung disease. Rheumatology.

[32] Shimizu T, Koga T, Furukawa K, Horai Y (2020). IL-15 is a biomarker involved in the development of rapidly progressive interstitial lung disease complicated with polymyositis/dermatomyositis. J Intern med.

[33] Moghadam-Kia S, Oddis CV, Sato S, Kuwana M, Aggarwal R (2016). Anti-melanoma differentiation–associated gene 5 is associated with rapidly progressive lung disease and poor survival in US patients with amyopathic and myopathic dermatomyositis. Arthr Care Res.

[34] Hall JC, Casciola-Rosen L, Samedy L-A (2013). Anti-melanoma differentiation-associated protein 5-associated dermatomyositis: Expanding the clinical spectrum. Arthritis Care Res.

[35] Ceribelli A, Fredi M, Taraborelli M, Cavazzana I, Tincani A, Selmi C (2014). Prevalence and clinical significance of anti-MDA5 antibodies in European patients with polymyositis/dermatomyositis. Clin Exp Rheum.

[36] Marie I, Hatron PY, Dominique S, Cherin P, Mouthon L, Menard J-F (2012). Short-term and long-term outcome of anti-Jo1-positive patients with anti-Ro52 antibody. Semin Arthritis Rheum.

[37] La Corte R, Lo Monaco A, Locaputo A, Dolzani F, Trotta F (2006). In patients with antisynthetase syndrome the occurrence of anti-Ro/SSA antibodies causes a more severe interstitial lung disease. Autoimmunity.

[38] Shi J, Li S, Yang H, Zhang Y, Peng Q, Lu X, Wang G (2017). Clinical profiles and prognosis of patients with distinct antisynthetase autoantibodies. J Rheumatology.

[39] Sabbagh S, Pinal-Fernandez I, Kishi T, Targoff IN, Miller FW, Rider LG (2019). Anti-Ro52 autoantibodies are associated with interstitial lung disease and more severe disease in patients with juvenile myositis. Ann Rheum Dis.

[40] Betteridge Z, Tansley S, Shaddick G, Chinoy H, Cooper RG, New RP (2019). Frequency, mutual exclusivity and clinical associations of myositis autoantibodies in a combined European cohort of idiopathic inflammatory myopathy patients. J Autoimmun.

[41] Spielmann L, Nespola B, Severac F, Andres E, Kessler R, Guffroy A (2019). Anti-Ku syndrome with elevated CK and anti-Ku syndrome with anti-dsDNA are two distinct entities with different outcomes. Ann Rheum Dis.

[42] Shappley C, Paik JJ, Saketkoo LA (2019). Myositis-related interstitial lung diseases: diagnostic features, treatment, and complications. Curr Treatm Opt Rheumatol.

[43] Perelas A, Silver RM, Arrossi AV, Highland KB (2020). Systemic sclerosis-associated interstitial lung disease. Lancet Respir Med.

[44] Marie I, Josse S, Hatron PY, Dominique S, Hachulla E, Janvresse A (2013). Interstitial lung disease in anti-Jo-1 patients with antisynthetase syndrome. Arthritis Care Res.

[45] Hobbs BD, Putman RK, Araki T, Nishino M, Gudmunsson G, Gudnason V (2019). Overlap of genetic risk between interstitial lung abnormalities and idiopathic pulmonary fibrosis. Am J Respir Crit Care Med.

