# Supplementary material for: Predictive Features and Clinical Presentation of Interstitial Lung Disease in Inflammatory Myositis
Source: Clin Rev Allergy Immunol. 2020 Nov 3;60(1):87–94. doi: 10.1007/s12016-020-08814-5 (PMC7819919; doi:10.1007/s12016-020-08814-5)
Supplement: Supplementary file 1 — Supplementary file1 (DOCX 12.7 kb) [file 12016_2020_8814_MOESM1_ESM.docx]

Table 1 (supplemental material): respiratory functional test in 15 patients with NSIP and 6 patients with UIP-like HRCT pattern

|  | NSIP n.15 (%) | UIP-like n.6 (%) | p |
| --- | --- | --- | --- |
| Onset DLCO, %, mean (SD) | 45.46 (9.6) | 73.17 (20.6) | 0.0004 |
| Onset DLCO/VA, %, mean (SD) | 75.54 (20.7) | 89.67 (23.6) | 0.189 |
| Onset FVC, %, mean (SD) | 74.69 (15.2) | 95.75 (23.5) | 0.02 |
